# Meta-Analysis of Factors Associated with Occupational Therapist Burnout

**DOI:** 10.1155/2021/1226841

**Published:** 2021-12-14

**Authors:** Eun-Young Park

**Affiliations:** Department of Secondary Special Education, College of Education, Jeonju University, P.O. Box 55059, 45 Baengma-gil, Wansan-gu, Jeonju, Republic of Korea

## Abstract

Burnout, a reaction to chronic emotional stress, affects health and reduces the quality of service. Reportedly, healthcare professionals are especially vulnerable to burnout. This meta-analysis is aimed at examining the factors associated with occupational therapists' burnout. The results of 2,430 occupational therapists, across 17 peer-reviewed English articles, the most recent published in 2020, were analysed. Results revealed significant associations between related variables and burnout. Marital status, work field, and work hours, job challenges, patient age, position, turnover intention, working type, and work addiction showed significant positive correlation effect sizes in relation to burnout, whereas age, education, engagement, job satisfaction, personal identity, professional identity, rewards, and feeling valued showed significant negative correlation effect sizes. The results of this meta-analysis suggest that strategies to reduce occupational therapists' burnout need to consider organizational as well as psychological aspects.

## 1. Introduction

As the quality of services provided in hospitals and the demand for patient satisfaction increase, so are the organizational efforts to control emotional expression when providing services. As a result, such efforts can bring about conflict between the emotional labour of the members of the organization and the role they play and may lead to burnout by reinforcing a negative attitude toward the job and organization [1–3]. Burnout refers to the state of psychological exhaustion experienced by individuals in jobs where they maintain long-term relationships with others and are exposed to prolonged fatigue, hostility, discouragement, maladjustment, discomfort, and restraint [4]. Individuals' continuous experience of negative emotions toward themselves and their work causes emotional depletion, depersonalization, and the overall reaction of a diminished sense of achievement [5].

Healthcare professionals are reported to be particularly vulnerable to burnout [6, 7]. Occupational therapists experience high levels of work-related job stress, as they require specialized knowledge and individualized treatment approaches for treatment interventions, communication, and teamwork between specialized areas for team access and continuous contact with patients targeted for treatment services [8]. Burnout is a reaction to chronic emotional stress, which affects health and reduces the quality of service [9–11]. In addition, it causes various problems such as absenteeism and turnover, lower productivity, and lower job satisfaction and organizational commitment, which are important components of job attitude [12–14].

Psychological exhaustion among occupational therapists is a critical problem that necessitates counteractive measures because it threatens their psychological well-being and is difficult to reverse. Therefore, it is important to develop preventive measures to reduce the incidence of burnout and its promotional factors in order to prevent individuals from becoming psychologically exhausted [15]. Studies related to burnout among occupational therapists are continuously being reported. Escudero-Escudero et al. [16] assessed the potential levels of burnout syndrome and risk factors in occupational therapists in Spain and found that 69.4% presented burnout syndrome and especially emotional fatigue (63.5%). They reported age, marital status, number of children, work field, and type of workday as risk factors. In Turkey, 26% of occupational therapists showed burnout symptoms, 38% were at risk, and working conditions, extrinsic and overall job satisfaction, vigour, and dedication accounted for 43.1% of the variance in burnout [17].

However, since it is difficult to present comprehensive results on the factors that affect burnout through a single study, it is necessary to synthesize and analyse the results of previous studies related to burnout among occupational therapists. For this reason, findings from different studies on burnout are often reported together. For instance, Park and Shin [18] performed a meta-analysis to verify associations between special education teachers' burnout and related variables. Kansoun et al. [19] reviewed a total of 37 studies to assess the prevalence of burnout and associated factors in French physicians and reported that the prevalence estimate of burnout was 49% and 5% for severe burnout.

Such studies are suitable for presenting implications according to the variables investigated in the study, such as gender, age, and career; however, they are insufficient for a comprehensive and systematic scientific verification of the variables that affect the burnout of occupational therapists. In addition, individual studies are conducted in different populations, using different tools, sampling methods, and statistical methods, and the characteristics of these individual studies may result in association found in these individual studies may contradict each other. For example, previous studies on the effect of gender on possession showed conflicting results, and for this reason, Purvanova and Muros [20] conducted a meta-analysis of 183 individual studies to analyse gender-based differences in burnout. Nevertheless, no meta-analysis investigating burnout in occupational therapists has been conducted so far.

Meta-analysis is a research method that is aimed at analysing and integrating several individual studies conducted on the same topic [21]. It uses quantitative and statistical methods to extract meaning by integrating the results of a number of studies implicitly, and its purpose is to draw universal and general conclusions from fragmentary results. The characteristics of meta-analysis are as follows: presentation of quantitative results, calculation of effect size through analysis of different research results, and derivation of integrated conclusions [22]. In other words, meta-analysis allows to synthesize prior studies in a systematic way, and as a research methodology to overcome the limitations of previous studies, it can play a complementary role to the existing literature. Since studies related to occupational therapists' burnout are being reported steadily, it is thought that the use of meta-analysis can provide integrated data on factors that affect burnout in this population. If the factors influencing the burnout of occupational therapists can be synthesized through meta-analysis, they can provide basic data for programs and policy suggestions to reduce occupational therapist burnout. Therefore, the purpose of this study was to identify the factors that influence burnout among occupational therapists through a meta-analysis.

## 2. Materials and Methods

### 2.1. Article Search and Selection Procedures

**Figure 1:**
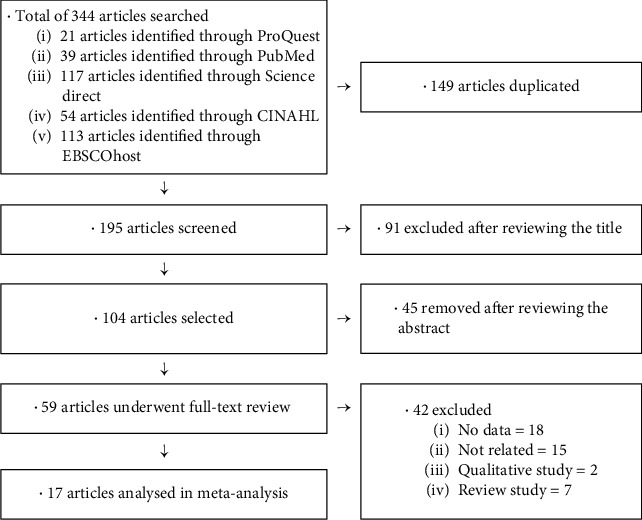
Article selection process.

The articles to be included in the meta-analysis of factors affecting burnout among occupational therapists were selected through a search of electronic databases ProQuest, PubMed, CINAHL, EBSCOhost, and Science Direct (December, 2020). The keyword combination “occupational therapist” and “burnout” was used. Through this process, a total of 344 articles were identified, which were then reviewed according to the following selection criteria: first, study participants were occupational therapists; second, the dependent variable was burnout; third, article was published in academic journals; fourth, article provided necessary data for calculating the effect size; fifth, article was published in English. The article selection process is presented in Figure 1.

### 2.2. Coding

The coding variables included author, publication year, burnout, burnout-related variables, participant characteristics (e.g., gender, age, education level, and experience), and burnout measurement tools. To check the direction of coding in each article, a coding standard was set, and articles with a different direction from the standard were recoded. The coding was as follows: gender, male “0”, female “1”; position, staff “0”, supervisor “1”; work field, small hospital “0”, large hospital “1”; marital status, unmarried “1”, married “0”; working time, full-time “0”, part-time “1”. Variables with only one effect size, specifically recession effect, control, community, fairness, and problem-solving, were coded as other. The three subdomains of burnout were coded for Maslach Burnout Inventory (MBI), and others were coded as two or more subdomains. A research assistant and the researcher performed coding for five articles (31.25% of the articles to be analysed), and the reliability between the coders was calculated for each coding variable. Reliability was calculated by dividing the coincidence frequency by the sum of the coincidence and disagreement frequencies and multiplying by 100. The reliability between the coders was 93.75% to 100%. Consensus was reached through discussions between researchers on inconsistent variables. One study, namely, Kim et al. [15], reported on physical and occupational therapists' burnout, and the data from occupational therapists were extracted.

### 2.3. Methodological Quality Assessment

The Quality Assessment and Validity Tool for Correlational Studies developed by Estabrooks et al. [23] was used to evaluate the quality of the articles. The evaluation items were as follows: 1 design question, 5 sample questions, 5 measurement questions, and 2 statistical analysis questions (total: 13 questions). Among the detailed items, the score for the use of a tool with an internal reliability of 0.70 or more for the dependent variable was 0 or 2 points, and other items were evaluated as 0 or 1. Since this study included group studies in addition to correlation studies, regarding the item on whether correlation analysis was applied to multiple outcomes, “correlation analysis” was modified to “appropriate statistical analysis.” The criteria for determining the overall study validity rating were as follows: “low” for 0–4 points, “medium” for 5–9 points, and “high” for 10–14 points. Among the 17 articles included in analysis, 8 were of high quality (47.1%), while 9 were of medium quality (52.9%).

### 2.4. Outlier and Publication Bias Test

Since extreme effect size values can cause a decrease in the reliability of the effect size, in this study, outliers were analysed to calculate a reliable effect size, and the effect size showing an outlier was excluded from the analysis. Outliers were evaluated through a review of residuals, and the criterion for judgment was the standardized residual value, *Z*, exceeding 2.58 (99% confidence level). There were no outliers with residuals above 2.58, and all effect sizes were thus included in further analyses.

To verify the publication bias, Rosenthal [24] evaluated the fail-safe *N*, which is aimed at establishing the stability of meta-analysis results, and examined the funnel plot. At a significance level of 0.05, the fail-safe *N*, which refers to the number of articles to be added for the effect size to be meaningless, was 228. Rosenthal [24] suggested the criterion value for judgement be 5 times the number of studies plus 10. In this study, 17 articles were selected for analysis, and if the stability factor was 95 or more, it could be considered to have no publication convenience. Egger's regression intercept was not significant (*p* = 0.78), which indicated absent publication bias.

### 2.5. Heterogeneity Test

To calculate the effect size, an effect size calculation model should be selected. There are two calculation models, a fixed effect model and a random effect model. Depending on which model is selected, the estimation and the precision of the effect size vary. The choice of model depends on whether the articles to be analysed are heterogeneous or homogeneous. In this study, a heterogeneity verification test was used, and the *p* value derived through this test was used as a reference value for the test of heterogeneity [25]. The result of *Q*-test yielded a *Q* value of 1701.075 (*p* < 0.001), which means that the included studies in this meta-analysis were heterogeneous. Based on these results, a random effect model was employed in this study.

### 2.6. Effect Size Calculation

The first step in a meta-analysis that presents the aggregated results of individual research results is the calculation of the effect size for each individual study. In the individual studies related to burnout among occupational therapists, there were cases in which the applied study design involved the comparison of burnout between groups (for example, the difference in burnout according to gender) and cases where a correlation coefficient was derived (for example, correlation between gender and burnout). To present comparison results between groups and for synthesizing all related research results, the standardized mean difference effect size was first calculated, and then, Fisher's *Z*-transformation was performed [22]. The effect size calculated in the meta-analysis was interpreted as small, if it was less than 0.1, medium, if it was between 0.10 and 0.49, or large, if it was greater than 0.5 [26].

## 3. Results

### 3.1. Characteristics of Individual Studies

The characteristics of the 17 included studies are described in Table 1. Altogether, 2,430 occupational therapists participated in surveys on burnout across the included studies. Three studies (17.6%) reported no specific numbers, only percentages regarding the gender of the occupational therapists [15, 27, 37].

Samples' ages generally ranged from 20 to 60 years; however, five studies did not report age, and four studies reported the mean age of their samples (32.0–44.5 years). Samples possessed an extensive range of years of experience, between 0 and 28 years, with four studies reporting only the mean years of experience (7.5–17.4) and three studies reporting no data on experience. Out of the 17 studies, 3 reported only one burnout-related variable, while the rest reported two or more related variables.

### 3.2. Effects of Related Variables

The overall effects of related variables on burnout are presented in Table 2. The variables with significant positive associations with burnout were job challenges (Fisher′s *Z* = 0.452, 95%CI = 0.307–0.597), marital status (Fisher′s *Z* = 0.075, 95%CI = 0.024–0.125), patient age (Fisher′s *Z* = 0.132, 95%CI = 0.074–0.191), position (Fisher′s *Z* = 0.196, 95%CI = 0.107–0.284), turnover intention (Fisher′s *Z* = 0.429, 95%CI = 0.297–0.562), working type (Fisher′s *Z* = 0.294, 95%CI = 0.140–0.448), work addiction (Fisher′s *Z* = 0.239, 95%CI = 0.179–0.298), work field (Fisher′s *Z* = 0.060, 95%CI = 0.021–0.098), and working hours (Fisher′s *Z* = 0.071, 95%CI = 0.007–0.134). Among the positive significant effect sizes, the effect size of marital status, work field, and work hours was small, and that of job challenges, patient age, position, turnover intention, working type, and work addiction was medium. The variables with significant negative effects on burnout were age (Fisher′s *Z* = −0.167, 95%CI = −0.219–−0.116), education (Fisher′s *Z* = −0.143, 95%CI = −0.232–−0.053), engagement (Fisher′s *Z* = −0.254, 95%CI = −0.419–−0.089), job satisfaction (Fisher′s *Z* = −0.462, 95%CI = −0.562–−0.362), personal identity (Fisher′s *Z* = −0.160, 95%CI = −0.211–−0.109), professional identity (Fisher′s *Z* = −0.405, 95%CI = −1.129–−0.082), and feeling valued (Fisher′s *Z* = −0.213, 95%CI = −0.268–−0.159). All negative significant effect sizes were medium.

## 4. Discussion

This study is aimed at verifying the effect size of variables related to occupational therapists' burnout through a meta-analysis. To achieve this, quantitative relations between burnout and its associated variables reported in previous studies were analysed.

Occupational therapists' burnout has been the subject of several studies, and many factors including challenges to professional identity, the emotionally demanding nature of mental health practice, lack of professional mentoring, and a lack of awareness of the value of the profession have been presented [35, 39, 40]. The association of burnout with personal (e.g., age and education level), psychological (e.g., job satisfaction, turnover intention, and identity), and organisational variables (e.g., reward, work field, and working type) have been verified by several previous studies [15, 17, 28, 40].

Overall, marital status (unmarried), work field (large hospital), work hours, job challenges, patient age, position, turnover intention, working type (part-time), and work addiction were associated with a higher level of occupational therapists' burnout. Conversely, age, experience, education, engagement, job satisfaction, personal identity, professional identity rewards, and values were associated with a lower level of occupational therapists' burnout.

Significant associations among personal variables were with marital status, position, age, and education, although there was no large effect size. Maslach and Jackson [41] reported that married therapists are generally older and psychologically more stable than unmarried ones because they have more experience in emotional conflict. Moreover, they found that their degree of burnout was lower than that of unmarried therapists because they received greater emotional support from their families. The positive significant effect size of marital status in this meta-analysis implies that burnout in married occupational therapists was lower than in unmarried ones, as the married group was the reference category in included studies. Since unmarried occupational therapists are highly likely to experience burnout, as discussed above, providing a support system, such as colleagues' support in the workplace, could alleviate their burnout levels. Regarding patient age, although Reis et al. [30] found that higher patient age increased the burnout rate, further research is required to confirm this relationship. On the other hand, Brewer and Shapard [42] reported that experience had a small negative relation with emotional exhaustion in their meta-analysis. However, the results of this meta-analysis showed that experience had no relation to burnout, among occupational therapists. One possible explanation is that occupational therapy is a health profession with high emotional labour intensity. It requires both physical effort and suppression and control of emotions for rehabilitation treatment, while providing rehabilitation treatment services to various patients and caregivers [43]. The job characteristics of occupational therapy can be inferred from the possibility that experience did not have an effect on reducing burnout.

Additionally, significant associations among the organisation-related variables were with patient age, work field, work hours, working type, and reward. The effect size of reward was medium, while the others were small. Reward refers to the compensation that employees receive for the time and effort given to the organisation [44]. The effort-reward imbalance model proposed by Siegrist [45] is considered a very influential theory in the field of occupational health, and the appropriateness of the theoretical model has been verified in several empirical studies targeting various occupational groups. This model, based on the norm of reciprocity, shows that the imbalance between effort and reward is related to health problems among members; a lack of reciprocity between effort and reward can cause job stress and lead to negative phenomena such as emotional exhaustion and work avoidance. Therefore, the balance between effort and reward (being sufficiently rewarded for the effort) is important for the well-being of organisational members. In previous studies, low reward has been reported to increase the likelihood of burnout [46–48]. A study examining the relationship between burnout and reward for occupational therapists also reported that inadequate reward was correlated with high burnout [49]. Next, regarding work field, stress in large hospitals was found to be higher than in small hospitals [50], which is consistent with the results of the present study. The relationship between work overload and burnout has also been reported by previous studies [2, 51]. In a study on burnout of nurses, the burnout of nurses working in small and medium hospitals was reported at a high level, as compared to large hospitals, due to the greater number of responsibilities [52]. Similarly, studies on occupational therapists also found that the size of hospitals had an effect on burnout, which indicates the necessity of in-depth research on the causes of these effects. Regarding work hours, Reis et al. [30] reported that occupational therapists who were working part time were more likely to experience higher burnout than those who were working full time; this corresponded with the results of this meta-analysis. In the study of variables related to organisation, the effect of the reward imbalance of effort with low reward compared to high effort on burnout has been reported [53]. Since an appropriate compensation system is a protective factor against burnout, the current occupational therapist's compensation system must be closely reviewed and revised accordingly.

In addition, significant associations among the psychological variables were with job challenges, turnover intention, work addition, engagement, job satisfaction, and personal value; the effect sizes of job challenges, job satisfaction, professional identity, and turnover intention were medium. Research on job challenges seems to have yet to reach conclusions. While job challenges can be positively related to ill-health (stress and burnout) as well as well-being (motivation and job satisfaction), as they lead to lower commitment to achieve goals, they also demand satisfaction, which motivates employees to work hard [54]. The results of this meta-analysis showed a positive relation between job challenges and burnout in occupational therapists. Members who experienced role conflict also had a high burnout rate [2, 55]. Intentional turnover as the primary outcome of burnout has been empirically supported by several studies [18, 56, 57]; however, the number of effect sizes for the relationship between burnout and turnover intention of occupational therapists was only two in this meta-analysis. Even though the relationship between turnover intention and burnout had a significant effect size, additional research is considered necessary. Further, numerous studies have reported a link between high burnout and low job satisfaction [58–60]. Although the correlation is high, the two variables are not necessarily of the same composition. This study found that occupational therapists' job satisfaction was linked to a positive perception of the therapeutic effect, associated with productivity and efficiency in their treatment behaviour; low job satisfaction also showed effects of increased helplessness and loss of motivation experienced in the job. An important focus of research on burnout reduction has been on interventions that improve the individual's ability to cope with various stressors in the workplace [61]. These individual abilities can include personal and professional identity. Given that occupational therapy is based on a core belief that participation in meaningful jobs promotes health and well-being [62], the relationship between professional identity and burnout is predictable. Work addiction, a constituent concept related to stress and burnout [63], has been defined as an uncontrolled motivation for work and the use of excessive energy and effort at work [64]. The nature of work addiction suggests a positive relationship with burnout, and the significant positive effect size in this meta-analysis supports this belief and the results of previous studies. Moreover, job engagement, expressed in terms of energy, participation, and efficacy, comprises the positive opposites of the three dimensions of burnout [65]. A meta-analysis study on the constructs of burnout and engagement also reported a high correlation between the two concepts [66]. The strong relationship between occupational therapists' engagement and burnout suggests that an increase in engagement has the potential to reduce burnout. Finally, feeling valued, namely, being flexible and autonomous from supervisors, respected as an occupational therapist, and able to use occupational therapy skills, was related to job satisfaction [14, 67, 68]. The negative relation between value and burnout in the present meta-analysis is considered to support previous studies.

## 5. Conclusions

The strength of this study was first a meta-analysis of the factors associated with burnout among occupational therapists. Among the personal variables related to burnout, those that had a positive association were marital status and position, and those with a negative association were age, education, and experience. All of the organisation-related variables, except for reward, were found to be associated with high level of burnout. Among the psychological variables, higher job challenges, work addition, and turnover intention were found to be associated with high level of burnout, while higher levels of engagement, job satisfaction, personal and professional identity, and feeling valued were found to be associated with low level of burnout. Among the related variables, psychological variables showed a relatively large effect size. As can be seen from the results, among the variables that affect burnout in occupational therapists, the number of personal variables was small and the effect size was also relatively small, whereas the number of related organisational and psychological variables and their effect size were relatively large. These results suggest that a strategy to reduce burnout in occupational therapists is needed. Occupational therapists' burnout can affect not only their quality of life but also the quality and effectiveness of treatment services. It is expected that intervention programs and policies to reduce the burnout of occupational therapists can be designed considering the factors found to be significantly associated with burnout among this population.

## Figures and Tables

**Table 1:** Characteristics of individual studies.

Author (year)	*N*	Age years (*n*)	Gender	Education level	Years of experience	Related variables	Measure	Country
Brollier et al. [27]	135	Mean = 32.0	NR	NR	Mean = 7.5	Position, work field, reward	MBI	USA
Rogers and Dodson [28]	109	Mean = 33.3	97 F, 12 M	62% = bachelor, 17% = PC, 14% = master, 7% = doctoral	Mean = 9.1	Age, education level, experience, position, patient contacts, religiosity		USA
Escudero-Escudero et al. [16]	758	<30 (274), 30-39 (351), above 40 (133)	688 F, 70 M		<1 = 87, 1‐2 = 225, 3‐5 = 128, 6‐8 = 98, above 12 = 103	Gender, age, marital status, number of children, work field, experience, patient contacts, working hours	MBI	Spain
Delos Reyes [29]	57	24-29	75% F, 25% M	74% = bachelor, 16% = during master, 9% = master, 1% = doctor	<2 = 42%, 3‐5 = 26%	Age, education level, experience, working hours, gender, marital status, working type, work field	MBI	Philippines
Reis et al. [30]	374	NR	344 F, 30 M	315 = licensed, 50 = master, 9 = bachelor	≤5 = 191, 6‐10 = 70, 11‐15 = 39, 16‐20 = 27, 21‐25 = 34, >25 = 13	Patient age, experience	MBI	Portugal
Kim et al. [15]	109	NR	NR	NR	NR	Work field	MBI	South Korea
Scanlan and Still [14]	113	<25 (16), 25-35 (44), 35-45 (19), >45 (22), mis (2)	94 F, 7 M, 2 mis	29 = PC, 29 = no PC, 2 = missing	<5 = 36, 6‐10 = 25, 11‐15 = 17, 16‐20 = 6, 21‐25 = 5, >25 = 14	Work/life balance, workload, reward, turnover intention	MBI	Australia
Gupta et al. [31]	53	M = 40.21	58 F, 5 M	Diploma = 3, bachelor = 39, master = 21	Mean = 14.72	Workload, control, reward, community, fairness, values	MBI	Canada
Brown and Pashniak [32]	140	20‐30 = 32, 31‐40 = 33, 41‐50 = 42, 51‐60 = 24, 60 ≤ 12	133 F, 6 M, 1 mis	Bachelor = 70, master = 49, PhD = 1	0 = 2, <1 = 3, 1‐4 = 32, 5‐9 = 21, 10‐14 = 19, 15‐19 = 19, 20 ≤ 47	Work addition	MBI	Canada
Scanlan and Hazelton [33]	118	<25 = 13, 35‐34 = 65, 35‐44 = 28, 45‐55 = 8, 55 ≤ = 4	110 F, 8 M	NR	<1 = 11, 1‐2 = 14, 2‐5 = 26, 5‐10 = 31, 10‐20 = 25, 20 ≤ 11	Job satisfaction, personal identity, values, patients contacts	OBI	Australia
Derakhshanrad et al. [34]	100	≤29 = 68, 30‐39 = 22, ≥40 = 10	58 F, 42 M	Most bachelor degree	NR	Problem-solving, gender, experience	MBI	Iran
Edwards and Dirette [35]	126	Mean = 44.5	91.3% F	Bachelor = 76.2%, master = 23.8%	Mean = 17.4	Professional identity	MBI	USA
Devery et al. [36]	10	NR	10 F	NR	NR	Job satisfaction, professional identity, job challenges	OBI	Australia
Anyfantis et al. [37]	247	20‐40 = 194, 41‐50 = 53	205 F, 42 M	NR	NR	Work field, age, marital status, recession effect, workload, experience	OBI	Greek & Cypriot
Abaoğlu et al. [17]	50	20‐25 = 25, 25‐30 = 23, 30‐35 = 2	40 F, 10 M	Bachelor = 30, under master = 10, master = 3, under PhD = 7	<1 = 4, 1‐2 = 10, 2‐3 = 14, 3 − 4 = 14, 4‐5 = 8	Job satisfaction, engagement, workload	BM	Turkey
Scanlan and Still [14]	34	<30 = 16, 31‐40 = 9, 41‐50 = 5, ≥50 = 3, mis = 1	27 F, 7 M	NR	1‐2 = 9, 2‐5 = 9, 5‐10 = 5, 10‐20 = 9, >20 = 2	Job satisfaction, turnover intention, workload, reward	OBI	Australia
Janus et al. [38]	97	Mean = 34	83 F, 14 M	Bachelor = 34%, master = 41%, vocational school = 2%, upper − secondary vocational education = 23%	<9 = 64%, 9‐14 = 25%, 19‐27 = 10%, 27 ≤ 1	Experience	MBI	Poland

Note. NR: not reported; M: male; F: female; mis: missing; PC: postbaccalaureate certificate; OBI: Oldenburg Burnout Inventory; BM: burnout measure; MBI: Maslach Burnout Inventory.

**Table 2:** Overall effect size of related variables on burnout.

Variables	*K*	-95% CI	ES	+95% CI	SE
Age	9	-0.219	-0.167	-0.116	0.026
Education	6	-0.232	-0.143	-0.053	0.046
Engagement	3	-0.419	-0.254	-0.089	0.084
Experience	25	-0.031	-0.049	0.033	0.042
Gender	5	-0.120	-0.036	0.048	0.043
Job challenges	26	0.307	0.452	0.597	0.074
Job satisfaction	7	-0.562	-0.462	-0.362	0.051
Marital status	8	0.024	0.075	0.125	0.026
Number of children	5	-0.101	-0.049	0.003	0.027
Other	5	-0.153	-0.065	0.024	0.045
Patient age	3	0.074	0.132	0.191	0.030
Patient contacts	7	-0.072	-0.021	0.030	0.026
Personal identity	2	-0.211	-0.160	-0.109	0.026
Position	6	0.107	0.196	0.284	0.045
Professional identity	12	-1.129	-0.405	-0.082	0.267
Religiosity	3	-0.130	-0.020	0.090	0.056
Reward	10	-0.310	-0.246	-0.182	0.033
Turnover intention	2	0.297	0.429	0.562	0.067
Values	12	-0.268	-0.213	-0.159	0.028
Working type	3	0.140	0.294	0.448	0.079
Work addiction	8	0.179	0.239	0.298	0.030
Work field	18	0.021	0.060	0.098	0.020
Working hours	5	0.007	0.071	0.134	0.032
Workload	8	-0.124	-0.063	0.002	0.031

Note. *K*: number of effect size; CI: confidence interval; ES: effect size; SE: standard error.
